# Renal Thrombotic Microangiopathy due to Hypertensive Emergency

**DOI:** 10.1155/crin/5096790

**Published:** 2025-07-16

**Authors:** Evan Perona, Matthew Kornas, Adrian G. Dumitrascu, Ricardo J. Pagan, Tatjana Gavrancic, Melissa P. Cortes, Aleksandra Murawska Baptista, Sam T. Albadri, Lyle W. Baker, Michael Smerina

**Affiliations:** ^1^Department of Internal Medicine, Mayo Clinic in Florida, Jacksonville, Florida, USA; ^2^Department of Laboratory Medicine and Pathology, Mayo Clinic, Rochester, Minnesota, USA; ^3^Department of Nephrology and Hypertension, Mayo Clinic in Florida, Jacksonville, Florida, USA

## Abstract

Thrombotic microangiopathy (TMA) is characterized by microvascular thrombosis, microangiopathic hemolytic anemia (MAHA), and thrombocytopenia. TMA can lead to acute kidney injury (AKI) due to the formation of thrombi within the renal microvasculature causing ischemic injury. AKI in the setting of TMA requires early recognition, comprehensive serologic evaluation, and timely intervention due to the risk of irreversible renal damage. Due to many potential causes, both hereditary and acquired, the workup of renal TMA includes analysis of ADAMTS13 activity, genetic testing, and antibody analysis to rule out extraneous etiologies. Ultimately, renal pathology is used to confirm the diagnosis. Recommended treatment of renal TMA is dependent on the underlying etiology and varies from therapeutic plasma exchange and anticomplement therapy to renal replacement therapy and supportive care. This case report highlights an underrecognized cause of renal TMA: hypertensive emergency. Pathological histology imaging of renal tubules can be used to diagnose renal TMA due to evidence of schistocytes and tubular necrosis. Diagnosing TMA can have life-saving consequences as delayed hemodialysis can be fatal. Renal pathological imaging should be an important diagnostic tool when presented with hypertension cases, especially those associated with the aforementioned symptoms. Blood pressure control is the primary focus for management of hypertensive emergency-associated TMA. We present a case of TMA-associated AKI in a hypertensive patient that had a characteristic onion-skin lesion seen on renal pathology.

## 1. Introduction

Hypertensive crisis is a rare but serious complication often attributed to inadequate hypertension management and prevention. Hypertensive crisis has been defined as the presence of a systolic blood pressure greater than 180 mmHg or a diastolic blood pressure greater than 120 mmHg. This phenomenon is further categorized into hypertensive urgency when end-organ damage is absent or hypertensive emergency when present [1]. Several different organ systems can be impacted in hypertensive emergency, with more common presentations including ischemic/hemorrhagic strokes, acute heart failure, or acute kidney injury (AKI). These complications can have devastating consequences for patients, with a review previously finding a close to 10% mortality rate among hospitalized patients with hypertensive emergency [2].

Due to the significant intravascular pressure in hypertensive emergency, smaller blood vessels can exhibit evidence of fibrinoid necrosis. This development has been associated with microangiopathic hemolytic anemia (MAHA) [1]—characterized by the destruction of red blood cells due to this intravascular disease. In addition, hypertensive emergency can be an underlying cause of thrombotic microangiopathy (TMA) [3–5]. TMA is defined by microvascular disease with intraluminal thrombus formation, MAHA, and thrombocytopenia [5, 6]. This disease process can cause end-organ damage via endothelial injury with subsequent activation of the coagulation cascade and complement system [5]. Secondary causes of TMA include systemic infections or cancer, autoimmune diseases such as scleroderma and systemic lupus erythematosus, and pregnancy-related conditions such as preeclampsia and HELLP syndrome [4]. As such, an extensive hematologic workup is necessary when TMA is suspected to refine the differential diagnosis—given that addressing the underlying disease is often the most effective treatment.

In this case, hemodialysis and aggressive blood pressure control were administered to a patient who presented with hypertensive emergency, kidney disease: improving global outcomes (KDIGO) stage III AKI, MAHA, and thrombocytopenia. Based on the evaluation and workup, it was determined that her AKI was induced by hypertensive emergency-related end organ damage. Previous literature has noted the formation of microthrombi and subsequent endothelial injury as a possible cause of AKI [4, 5, 7]. As such, the workup of this case and related pathological imaging is summarized in the following case presentation.

## 2. Case Presentation

A 42-year-old African American female with a history of hypertension, BMI of 24, active tobacco use, and tetrahydrocannabinol (THC) use presented to an outside facility with symptoms of nausea, abdominal pain, and vomiting. Blood pressure on presentation was 257/165 mmHg, meeting criteria for hypertensive crisis. Further workup revealed evidence of a stage III AKI based on KDIGO classification. In addition, the patient's hemoglobin was significantly decreased at 7.0 g/dL with a correspondingly increased schistocyte level at 20%, indicating the presence of hemolytic anemia such as MAHA. She was notably thrombocytopenic, and these results were subsequently confirmed on a peripheral smear.

The patient was transferred to a tertiary hospital for further management given concerns that she may require plasmapheresis and/or renal replacement therapy. Her hypertension was treated with infusions of nicardipine and bumetanide. Plasmapheresis was started given concerns for potential thrombotic thrombocytopenic purpura (TTP) and atypical hemolytic uremic syndrome (aHUS) while awaiting results of her serologic evaluation. Initiation of renal replacement therapy via hemodialysis was eventually required due to the patient's development of uremic symptoms.

Once the patient was temporized with plasmapheresis and hemodialysis, further workup was performed to refine the differential diagnosis (Table 1). Primary aldosteronism testing revealed that plasma aldosterone and renin activity were both elevated at 30 ng/dL and 37 ng/mL/h, respectively. An atypical HUS/TMA complement panel demonstrated normal Factor B, Factor H, C3, C4, and C4d levels. Only a mild elevation in SC5b-9 (soluble membrane attack complex) was identified. Genetic testing with a comprehensive complement genetic panel of 15 genes found no pathogenic variants or variants of uncertain significance. Serologic testing for antiphospholipid antibody syndrome (including phospholipid cardiolipin antibodies, lupus anticoagulant profile, and beta-2 glycoprotein antibodies) was negative. MRI brain imaging showed no evidence of posterior reversible encephalopathy syndrome (PRES).

Renal biopsy demonstrated mild chronic injury with focal global glomerulosclerosis (7 of 52 glomeruli), mild interstitial fibrosis, and tubular atrophy involving approximately 10%–20% of the cortical area. This evidence supported a diagnosis of pre-existing, likely subclinical, chronic kidney disease (CKD) prior to the patient's presentation. Findings of acute vascular injury included striking arteriolar hyalinosis with onion skinning, diffuse myxoid intimal edema of medium to large arteries, and intraluminal fibrin deposition. Collectively, biopsy findings were consistent with acute on chronic renal insufficiency.

**Figure 1:**
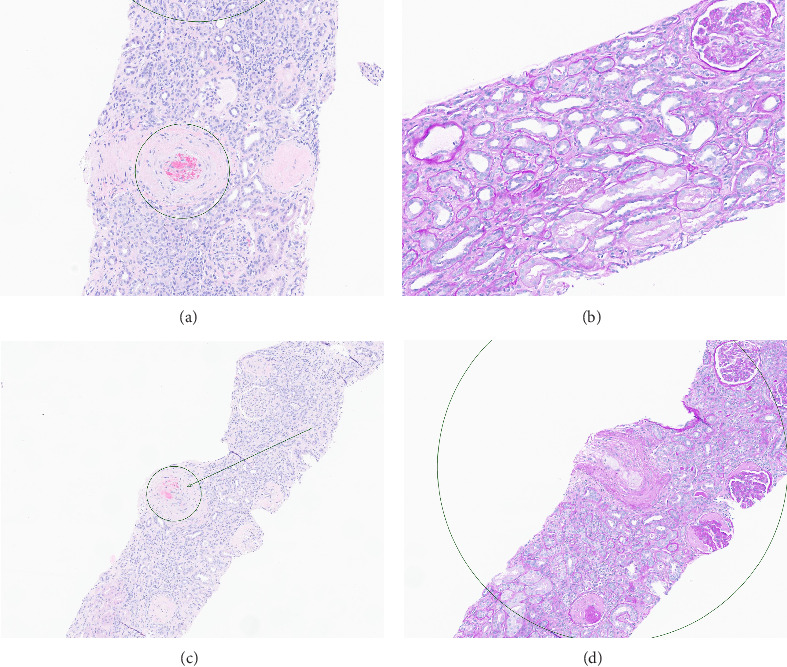
Pathological imaging of renal tubules. (a) Onion skin features of a medium-sized artery with fragmented red blood cells and fibrin within the arterial lumen. (b) PAS stain illustrating acute tubular injury. (c) Fragmented erythrocytes due to hypertensive crisis. (d) Increased prevalence of fragmented erythrocytes occluded with arterial mucoid foamy cells.

Based on this evidence, hypertensive emergency was identified as the root cause of the fragmented erythrocytes (schistocytes), fibroid necrosis, and acute tubular injury within the renal vasculature (Figure 1). The evidence of acute vascular injury on renal biopsy included features associated with hypertension-associated TMA. These findings are, therefore, consistent with the final diagnosis of TMA resulting from hypertensive emergency.

Over the course of the patient's hospitalization, hypertensive control was achieved with oral agents while weaning off infusion therapy. She was discharged on multiple antihypertensive agents including bumetanide, carvedilol, nifedipine, isosorbide mononitrate, and lisinopril for hypertension management. She was liberated from dialysis after two sessions and maintained adequate renal recovery before her discharge from the hospital. A monthly checkup revealed the patient to be asymptomatic with full adherence to this medication regimen.

## 3. Discussion

This case demonstrates a less common but clinically relevant case of hypertensive emergency as a secondary cause of TMA. The broad range of possible etiologies include TTP, aHUS, disseminated intravascular coagulation (DIC), preeclampsia, severe hypertension, autoimmune disease, and systemic infection and cancer [3–7]. Given these diverse pathologies, a comprehensive history and physical, extensive lab workup, and pathology evaluation are important tools to establish the underlying etiology and subsequent treatment.

CBC with differential should be obtained to assess for acute anemia, increased reticulocytes, acute thrombocytopenia, and presence of schistocytes, which will indicate hemolysis and thrombocyte consumption. Due to the hemolytic nature of this disease, assessment of LDH and haptoglobin will typically yield abnormal results [6]. Urine studies including urinalysis with microscopy should be performed to assess for red blood cells (RBCs), RBC casts, and proteinuria. Additionally for our patient, elevation of both plasma renin activity and aldosterone was not consistent with a diagnosis of primary aldosteronism. We postulate that the elevated renin level likely reflected pronounced renin–angiotensin system activation secondary to renal ischemia and severe hypertension.

TTP can be excluded with a normal range of ADAMTS13 activity [3, 4], and an aHUS/TMA/C3G gene panel can assist in ruling out genetic predispositions. Testing of our patient revealed an absence of complement regulatory abnormalities, which argued against an underlying complement-mediated TMA such as aHUS. Mild SC5b-9 elevation is a nonspecific finding that reflects terminal complement activation and may occur in a variety of TMAs—including those associated with hypertensive emergency. Additional serologic testing can reveal potential autoimmune or rheumatologic maladies. However, this patient's antibody testing for antiphospholipid syndrome and Goodpasture syndrome was negative. A negative Direct Coombs Test also can rule out immune-mediated hemolysis. Lastly, preeclampsia and HELLP syndrome were excluded given that our patient was not pregnant.

Of note, ADAMTS13 activity testing is labor intensive and not readily available at most institutions—meaning plasma exchange should be administered if TTP is reasonably suspected given its high mortality without treatment [8]. The PLASMIC score can be calculated to help guide this decision-making. This score incorporates multiple standard and readily available laboratory tests to stratify patients into low, intermediate, or high-risk categories for TTP [9]. Components include platelet count, creatinine level, evidence of hemolysis (high reticulocyte count and indirect bilirubin and undetectable haptoglobin), and other associated conditions (lack of active cancer, solid organ, or hematopoietic stem cell transplant) [9]. A high probability score of 6–7 supports the use of immediate plasma exchange, while a low probability score of 4 or less instead supports evaluation for other etiologies [9]. The patient in this case received empiric plasma exchange due to concern of TTP, with subsequent renal biopsy confirming a hypertensive etiology. She responded well to antihypertensive agents. Of note, these patients have been noted to have a higher incidence of persistent renal failure requiring hemodialysis compared with patients diagnosed with TTP [10, 11].

Hypertensive emergency has historically been associated with a poor prognosis, with more recent studies indicating significant inpatient mortality rates [2]. These outcomes are often due to the end organ damage associated with this condition, including cerebrovascular accidents, acute heart failure, and AKI [2]. While stabilizing the patient's organ injury and blood pressure, further assessment is important to help determine the appropriate treatment course based on the etiology of TMA. Cavero et al. demonstrated primary hypertension and glomerular diseases as frequent causes of hypertensive emergency but noted that primary aHUS and drug-induced hypertension (antitumoral drugs and cocaine) were seen in high proportions of TMA cases. In addition, among patients evaluated for malignant hypertension, TMA was identified in 2.3% of the cases after exclusion of alternative cases such as aHUS, drug-related TMA, systemic diseases, and glomerular diseases [11]. As such, malignant hypertension can be a less common yet independent cause of TMA. Kidney biopsy is also necessary to evaluate the renal histopathology and confirm a diagnosis of renal TMA. This imaging is critical in finding vasculature damage, necrosis, and erythrocyte fragmentation. Therefore, following a comprehensive evaluation, hypertensive emergency was determined to be the most probable cause of TMA for our patient case.

After acute treatment of hypertensive emergency, modifiable risk factors should be addressed to help prevent future uncontrolled hypertension and repeat acute organ damage. Previous reviews have noted multiple factors associated with the development of hypertensive crisis, including unhealthy alcohol use, recreational drug use, and a history of cardiovascular conditions such as coronary artery disease, congestive heart failure, and CKD [12]. In addition, risk factors associated with hypertensive emergency specifically have included diabetes, hyperlipidemia, and CKD [12]. This patient was reportedly noncompliant with her previous antihypertensive therapy prior to evaluation, contributing to this acute exacerbation. The patient's antihypertensive regimen was adjusted prior to discharge and subsequent follow-up helped ensure confirmation of compliance with her medication therapy.

Previous research in the field of THC has shown that marijuana has an inconsistent relationship with its impact on blood pressure. For example, it was found that cannabis use significantly increased systolic blood pressure in regular users but did not have the same impact on those same users' diastolic blood pressures [13]. Another study found that a decrease in systolic blood pressure, diastolic pressure, and pulse pressure was associated with regular THC users [14]. Further research will be needed to better elucidate the relationship between marijuana use and blood pressure, but it must remain a considered factor when part of a patient's history.

## 4. Conclusion

Overall, this case revealed that hypertensive crisis can present with TMA-induced AKI. Differential diagnosis and hematologic workup are critical for determining a secondary underlying etiology, including evaluation for TTP and aHUS, due to the difference of emergent indicated therapies. Proper diagnosis of renal TMA requires renal biopsies and pathological imaging to assess potential vasculature problems of scarring, blockages, and/or chronicity. Nonadherence to blood pressure medications may accelerate and exacerbate the consequences of hypertensive crisis episodes, including potential chronic renal insufficiency requiring hemodialysis. It is crucial to educate patients on the possible consequences of hypertension and the importance of medication adherence to improve patient outcomes.

## Figures and Tables

**Table 1:** Condition-specific measurements upon patient arrival versus normal values.

Condition	Patient value (normal range)
Blood pressure (mm Hg)	257/165 (120/30)
Leukocyte count (× 10°/L)	9.0 (3.4–9.6)
Erythrocyte count (× 10°/L)	2.28 (3.92–5.13)
Hemoglobin (g/dL)	(7.0 (11.6–15.0
Platelet count (× 10% L)	52 (157–371)
ADAMTS13 activity (%)	74 (270)
Lactate dehydrogenase (U/L)	314 (122–222)
aHUS/TMA/C3G gene panel	Negative (negative)
Schistocyte level (%)	20 (0–0.5)
Antinuclear antibody ratio	0.5 (0–1)
Sel 70 antibodies	0.2 (< 1)
Anti-dsDNA antibodies	12.3 (< 30)
Glomerular basement membrane antibodies	0.2 (< 1.0)
Direct Coombs test	Negative (negative)

*Note:* Units are located beside each specified condition.

## Data Availability

The data that support the findings of this study are available from the corresponding author upon reasonable request.

## Nomenclature

TMA: Thrombotic microangiopathy
MAHA: Microangiopathic hemolytic anemia
AKI: Acute kidney injury
KDIGO: Kidney disease: improving global outcomes
THC: Tetrahydrocannabinol
TTP: Thrombotic thrombocytopenic purpura
aHUS: Atypical hemolytic uremic syndrome
DIC: Disseminated intravascular coagulation
CBC: Complete blood count
LDH: Lactate dehydrogenase
CKD: Chronic kidney disease
